# Autonomous Vision-Based Aerial Grasping for Rotorcraft Unmanned Aerial Vehicles

**DOI:** 10.3390/s19153410

**Published:** 2019-08-03

**Authors:** Lishan Lin, Yuji Yang, Hui Cheng, Xuechen Chen

**Affiliations:** School of Data and Computer Science, Sun Yat-sen University, Guangzhou 510006, China

**Keywords:** autonomous aerial grasping, unmanned aerial vehicle, visual perception, localization

## Abstract

Autonomous vision-based aerial grasping is an essential and challenging task for aerial manipulation missions. In this paper, we propose a vision-based aerial grasping system for a Rotorcraft Unmanned Aerial Vehicle (UAV) to grasp a target object. The UAV system is equipped with a monocular camera, a 3-DOF robotic arm with a gripper and a Jetson TK1 computer. Efficient and reliable visual detectors and control laws are crucial for autonomous aerial grasping using limited onboard sensing and computational capabilities. To detect and track the target object in real time, an efficient proposal algorithm is presented to reliably estimate the region of interest (ROI), then a correlation filter-based classifier is developed to track the detected object. Moreover, a support vector regression (SVR)-based grasping position detector is proposed to improve the grasp success rate with high computational efficiency. Using the estimated grasping position and the UAV?Äôs states, novel control laws of the UAV and the robotic arm are proposed to perform aerial grasping. Extensive simulations and outdoor flight experiments have been implemented. The experimental results illustrate that the proposed vision-based aerial grasping system can autonomously and reliably grasp the target object while working entirely onboard.

## 1. Introduction

There is increasing interests in unmanned aerial vehicles (UAVs) within both the industrial and academic communities. Vertical takeoff and landing (VTOL) unmanned rotorcrafts with onboard lightweight visual sensors have broad applications including surveillance, monitoring, rescue and search, traffic control, etc. [1,2]. With the high 3-D mobility, UAVs act like smart flying cameras in passive observation applications. A UAV equipped with a robotic arm can perform aerial manipulation tasks like grasping, placing and pushing objects [3]. Integrating the high mobility of UAVs as well as the manipulation skills of robotic arms, UAVs mounted with robotic arms will actively interact with environments and have widely potential applications in transportation, building, bridge inspection, rotor blade repairing, etc. [4].

Vision-based aerial manipulation for micro UAVs poses challenges due to the inherent instability of the UAVs, limited onboard sensing and computational capabilities, and aerodynamic disturbances in close contact. Modeling and control, motion planning, perception, and mechanism design are crucial for aerial manipulations [5,6,7]. There are some challenges for UAVs to perform autonomous vision-based aerial grasping. These challenging problems mainly come from the following aspects: (1) the limitation imposed by the high-order underactuated control systems; (2) the limited onboard vision-based sensing; (3) highly computational efficiency of visual detection, estimation of grasping points of the target object, and control of the UAV equipped with a robotic arm are required for onboard implementation using a low-cost embedded controller; (4) coupling between perception and control of the aerial manipulation system.

Motived by the challenging problems, we systematically investigate a vision-based strategy to perform aerial grasping by an UAV. The contributions of this paper are presented as follows:

1. A new learning module is proposed for real-time target object detection and tracking. Concretely, the proposed scheme extends the kernelized correlation filters (KCF) algorithm [8] by integrating the frequency-tuned (FT) salient region detection [9], the K-means and the correlation filter algorithms, which is able to detect the target object autonomously before tracking without human involvement.
2. To increase the success rate of grasp, a computationally efficient algorithm based on support vector regression (SVR) is proposed to estimate appropriate grasping positions of the visually recognized target object.
3. A control strategy is proposed to perform aerial grasping, which consists of approaching and grasping phases. During the approaching phase, a nonlinear control law is presented for an UAV to approach the target object stably; while during the grasping phase, simple and efficient control schemes of the UAV and the robotic arm are presented to achieve the grasping based on the estimated relative position between the UAV and the target object.
4. A computationally efficient framework implemented on an onboard low-cost TK1 computer is presented for UAVs to perform aerial grasping tasks in outdoor environments. The proposed visual perception and control strategies are systematically studied. Simulation and real-world experimental results verify the effectiveness the proposed vision-based aerial grasping method.

The rest of the paper is organized as follows. Section 2 describes the related work. In Section 3, the system configuration is described. In Section 4, detection and recognition of target object, as well as an estimation of its grasping points, are proposed. The grasping strategy and control of the aerial grasping system is presented in Section 5. Experimental results are presented in Section 6. Concluding remarks and future work are discussed in Section 7.

## 2. Related Work

Aerial manipulation is a challenging task, and some of the pioneering works in this area appeared in the literature [10,11,12,13,14,15]. Visual perception, control and motion planning of UAVs, and mechanism design of the end-effector, are essential for an aerial manipulation system.

Real-time target object detection is vital to perform autonomous grasping of a target object. Currently, deep learning-based algorithms [16,17,18] achieve excellent detection performance, which usually require high computational complexities and power consumptions. However, the computational capacities of an onboard computer are limited due to the payload of the micro UAVs, and the deep learning-based approaches are not suitable for real-time aerial grasping. Traditional manual feature detection algorithms [19] are highly computational efficiency, but it is still not enough to run in real time on the low-cost onboard computer of an UAV.

Estimating grasping points of the target object is beneficial to improving the grasping performance. In [20], a target pose estimation algorithm is proposed to estimate the optimal grasping points using the manual threshold. Pose estimation helps to estimate the grasping points, but the manual threshold brings difficulties when applying it to various target objects. In [21,22,23], different markers are used to perform real-time target detection, while target objects cannot be detected in the absence of artificial markers. To guide the UAV to autonomously perform grasping of the target object, with the target object detection information, the relative position between the UAV and the target object should be continuously estimated to guide the motion of the UAV and the onboard robotic arm. In [24,25,26,27], various aerial grasping approaches are presented, where the relative position of the target object is obtained by high performance indoor positioning systems. It hinders the aerial grasping in environments without positioning systems.

Real-time target tracking need to be performed during the aerial grasping process. Discriminative correlation filter (DCF)-based approaches as well as deep learning-based methods [28] are two major categories of visual object tracking. The computational efficiency of the DCF-based approaches is much higher than that of the deep learning-based algorithms. In our previous work [29], the Kernelized Correlation Filter (KCF) tracker [8] is adopted for an UAV to track the moving target, where the object of interested region is chosen manually at the first frame. In this paper, the KCF tracker is applied for visual tracking of the autonomously detected target for its computational efficiency and impressive performance.

Stable control of the UAV is important for an aerial grasping system. In [21], the traditional PID controller is modified by adding nonlinear terms which usually require experimental or accurate measurements. The parameters of the proposed controller are difficult to set, also it is difficult to adapt the controller to different mechanical structures. In [24], a PID controller is employed for the UAV to follow the planned path. However, the parameters tuning of the PID controller is difficult for high-order underactuated UAV control systems. In this paper, a nonlinear and computationally efficient controller is proposed to guide the UAV stably approaching the target object based on the estimated relative position information.

In this paper, using onboard sensing and computational capabilities, we aim to investigate the problem to autonomous grasp the target object without manually choosing the object of interested region in advance. A visual-based aerial grasping scheme is presented, where computationally efficient approaches are proposed for target detection, grasping points estimation and relative position estimation. Moreover, efficient control laws are presented for the UAV and the onboard robotic arm to perform stably aerial grasping.

## 3. System Configuration

**Figure 1:**
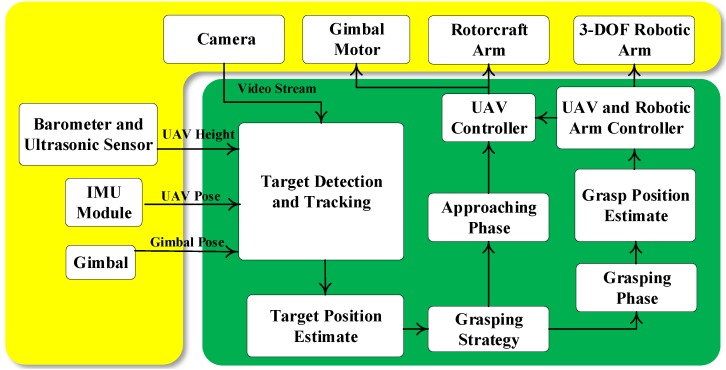
Architecture of the autonomous vision-based aerial grasping system.

Figure 1 illustrates the configuration of an autonomous vision-based aerial grasping system. The yellow box is the hardware part of the system, and the green box is the software part of the system. A DJI Matrice 100 is used as an experimental platform, which is equipped with a DJI Manifold embedded Linux computer, a monocular gimbal camera and a 3-DOF robotic arm. The gimbal camera provides the video stream for the embedded computer. The target object is detected, recognized and tracked in real time. The grasping points of the recognized target object are then estimated to increase the grasping success rate. To perform stably aerial grasp, using the relative position between the UAV and the target object, the grasping process is divided into the approaching and the grasping phases. In these two phases, different control strategies are developed for the aerial grasping system.

## 4. Vision System

In this section, a computationally efficient visual object detection and tracking scheme is presented to continuously locate the target position in the image. Moreover, a novel real-time algorithm is proposed to estimate the grasping positions of the target object to improve the grasping performance.

### 4.1. Object Detection

To reduce the computational complexity, the visual object detection scheme is separated into two steps, i.e., region proposal as well as classification. Firstly, all regions of interest (ROIs) are detected in the image using the region proposal algorithm. Then the target object in all ROIs is recognized with the designed classifier.

#### 4.1.1. Region Proposal Algorithm

Because of high computational efficiency in the Fourier domain, the Frequency-Tuned (FT) saliency detection [9] is adopted to obtain the saliency map, which can be used to extract ROIs. The quality of the image captured from the onboard gimbal camera is affected by factors such as illumination, unstable hovering of UAV and so on. It deteriorates the robustness of the method combining the FT and the K-means in outdoor applications. In this paper, an improved region proposal algorithm integrating by the FT and the K-means is presented.

Firstly, summing continuously *n* frames of the saliency map to obtain the cumulative image $I_{RSsum}$, i.e.,

(1) $$I_{RSsum}=\sum_{i=1}^{n}I_{RS_{i}},$$

where $I_{RS_{i}}$ is the output of the FT algorithm for the *i*th frame. Denote $I_{RSBW}$ the binarization of $I_{RSsum}$ as $I_{RSBW}$. $I_{RSBW}$ represent the contours and the centroids of the connected components, and are calculated to obtain the initial model of the current scene. The model $M_{s}$ is represented as

(2) $$M_{s}=\left\{I_{RSBW},C_{e},C_{c}\right\},$$

where $C_{e}$ are the contours of the connected components and $C_{c}$ are the centroids of the connected components. These steps are implemented repeatedly at every *n* frames of the saliency map. The old model $M_{s}$ of the current scene is updated with the new models at every *n* frames, and the convolution is used for the update. Specifically, K candidate contours in the new model are employed to update the old model by convolution. The candidate contours are chosen by the nearest neighbor between the new model and the old model. The contours and centroids are updated simultaneously according to

(3) $$M_{s}=\left\{M_{Si}⊗M_{SNewi}\cup \left\{M_{s}-\left\{M_{Si}\right\}\right\},i=1,\cdots ,K\right\}.\;$$

Define a set $B=\{C_{e},C_{c}\in M_{s}\}$ describing contours and centroids to denote the region of all possible target objects. Algorithm 1 describes the flow of the region proposal algorithm.

**Algorithm 1:** Region Proposal Algorithm
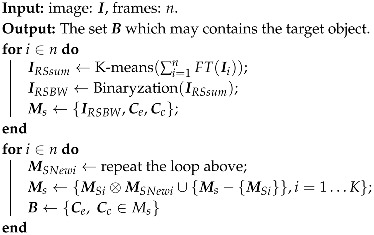


#### 4.1.2. Classification

**Figure 2:**
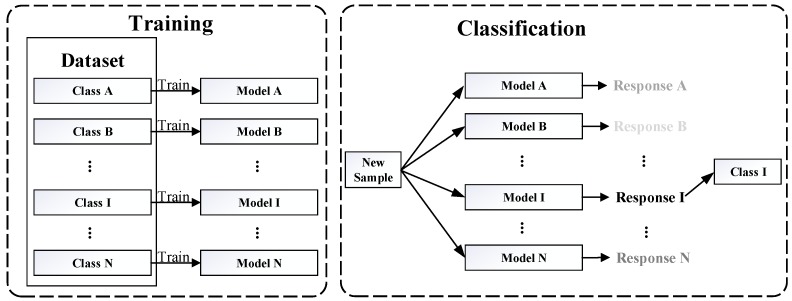
The training and classification process of the algorithm.

The computationally efficient KCF algorithm [8] is applied for tracking the target when it is detected. It is obvious that the efficiency of combination between the target detection and the KCF algorithm should be considered. Therefore, a KCF-based target classifier is presented in this section. The training and classification process of the algorithm are shown in Figure 2. The framework of the algorithm is similar to [30]. Firstly, we train a model in the same way for each class. These models are used to classify new samples. Response values represent the evaluation of new samples by these models. As shown in Figure 2, the depth of the font “response” color represents the strength of the response. For example, a new sample through model A∼N. The response I is the strongest response value, thus the new sample is classified to class I. The algorithm of classification is described as follows.

The KCF tracker learns a kernelized least squares classifier of a target. A classifier is trained using the RGB iamge patch *x* of size M×N that is centred around the target. The tracker considers all cyclic shifts $x_{m,n}$, (m,n)∈{0,…,M−1}×{0,…,N−1} as one training examples for the classifier. These are labelled with a Gaussian function *y*, so that y(m,n) is the label for $x_{m,n}$. The goal of training is to find a function $f\left(x_{m,n}\right)=\omega^{T}x_{m,n}$ to minimize the squared error over samples $x_{m,n}$ and their regression targets $y_{m,n}$,

(4) $$\min_{\omega }\sum_{m,n}{\left(f\left(x_{m,n}\right)-y_{m,n}\right)}^{2}+\lambda {∥\omega ∥}^{2},$$

where λ is a regularization parameter that controls overfitting, as in the Support Vector Machines (SVM) method [31].

Mapping the inputs of a linear problem to a non-linear feature-space ϕ(x) with the kernel trick, the ω can be calculated [32] by

(5) $$\omega =\sum_{m,n}\alpha \left(m,n\right)\phi \left(x_{m,n}\right),$$

where ϕ is the mapping to the a non-linear feature-space induced by the kernel κ, defining the inner product as $\langle \phi (f),\phi (g)\rangle =\kappa \left(f,g\right)$. In the meanwhile, $f\left(z\right)=\omega^{T}z=\sum_{i=1}^{n}\alpha_{i}\kappa \left(z,x_{i}\right)$. Thus, the variables under optimization are α, instead of ω. The coefficients α in Equation (5) can be calculated by

(6) $$A=F\left\{\alpha \right\}=\frac{Y}{U_{x}+\lambda },$$

where F is the DFT (Discrete Fourier Transform) operator, *Y* is the DFT of *y*, $U_{x}$ is the DFT of $u_{x}$ and $u_{x}=\kappa \left(f\left(x_{m,n}\right),f\left(x\right)\right)$ is the output of the kernel function κ.

For the off-line training, the model is trained according to Equation (6) for each sample. All models of one class are stitched into a vector:

(7) $$F={\left[f_{1},\cdots ,f_{i},\cdots ,f_{n_{p}}\right]}^{T},$$

where F is a filter vector whose element $f_{i}$ is a filter which obtained by training the *i*th sample, and $n_{p}$ is the number of samples.

Each filter $f_{i}$ is applied for evaluating the other positive sample by correlation operation beside the sample which is trained for itself. The evaluation matrix is shown below

(8) $$V_{response}=\left[\begin{array}{cccc} f_{1}\left(x_{2}\right) & f_{1}\left(x_{3}\right) & \cdots & f_{1}\left(x_{n_{p}}\right)\\ f_{2}\left(x_{1}\right) & f_{2}\left(x_{3}\right) & \cdots & f_{2}\left(x_{n_{p}}\right)\\ \vdots & \vdots & \ddots & \vdots \\ f_{n_{p}}\left(x_{1}\right) & f_{n_{p}}\left(x_{2}\right) & \cdots & f_{n_{p}}\left(x_{n_{p}-1}\right) \end{array}\right],$$

where $f_{i}\left(x_{j}\right)$ is the correlation evaluation of the *i*th sample and the *j*th sample.

There are $n_{p}-1$ evaluation values for each filter, and they can be written as a vector. All the elements of the vector are summed as the evaluation value for the filter. Thus, there are *n* filters so that the number of the evaluation values is *n*. Finally, all the evaluation values of each filter can be written as a normalized vector and all the elements of this vector are called the weight coefficient of the corresponding filter. Its vector form is

(9) $$C_{f}=\left[\begin{array}{c} \frac{1}{n_{p}-1}\left(\sum_{i\neq 1,i=2}^{n_{p}}f_{1}\left(x_{i}\right)\right)\to \left(0,1\right]\\ \frac{1}{n_{p}-1}\left(\sum_{i\neq 2,i=1}^{n_{p}}f_{1}\left(x_{i}\right)\right)\to \left(0,1\right]\\ \vdots \\ \frac{1}{n_{p}-1}\left(\sum_{i\neq n_{p},i=1}^{n_{p}}f_{1}\left(x_{i}\right)\right)\to \left(0,1\right] \end{array}\right],$$

Then the final model of target is written as:

(10) $$f_{cls_{n}}=C_{f}^{T}*F,n=1,2,\cdots ,n_{c},$$

Algorithm 2 describes the training flow of the correlation filter based on ridge regression.

**Algorithm 2:** The training algorithm of the KCF-based target classifier
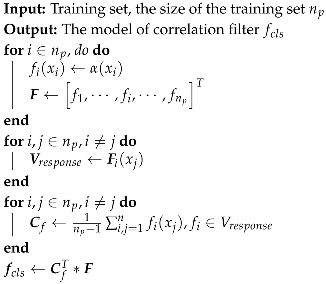


### 4.2. Grasp Position Estimation

In this section, a real-time estimation algorithm of the grasping position is presented based on support vector regression (SVR). A grasping position estimate is beneficial to improve the grasping performance because of the significant shape feature of the target object.

**Figure 3:**
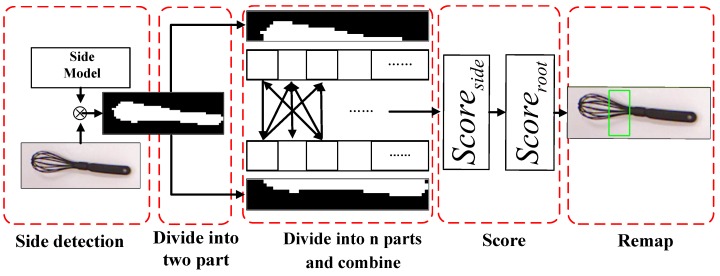
The flow of the grasping position detection algorithm.

Lenz et al. show that the feature of grasping position can be easily described by the depth image provide by the RGB-D camera [33]. However, the performance will degenerate greatly in outdoor environments as the RGB-D camera is accessible to the lighting interference. In this paper, RGB images are used for grasping position estimation because (1) the HOG features [19] can represent the magnitude and direction of the gradient at the same time, (2) the feature of symmetry is apparent in the HOG features, and (3) the consumption of computation in the HOG features can be ignored, the HOG features are extracted for grasping position estimation from RGB images. Figure 3 shows the flow of the grasping position detection algorithm.

According to the symmetry of gradient value and direction of the grasping point of the target, the model training can be divided into two parts, one part is to learn a root model from the whole points of the grasping position, while another part is to train a side model from the edge feature of the target object. The same training method is used for the root model and the side model.

The root and size models are denoted as S and R, respectively. They can be trained to optimize Equation (11) with SVR:

(11) $$\begin{array}{c} \min\frac{1}{2}{∥\omega ∥}^{2}+C\sum_{i=1}^{l}\left(\xi_{i}+\xi_{i}^{*}\right),\\ s.t.\left\{\begin{array}{c} y_{i}-\left(\omega^{T}+b\right)<\epsilon +\xi_{i},\\ \left(\omega^{T}+b\right)-y_{i}<\epsilon +\xi_{i}^{*},\\ \xi_{i},\xi_{i}^{*}>0, \end{array}\right. \end{array}$$

where *C* is the penalty factor, $\xi_{i}$ and $\xi_{i}^{*}$ are used to construct soft margin, and *l* is the number of the samples.

The HOG feature map of the input image, which is part of the whole image, is denoted as G. The edges information and the response map T about the shape information of the target object can be obtained as follows:

(12) $$\left\{\begin{array}{c} T=\eta \left(x,y\right)=\sum_{x^{\prime },y^{\prime }}S\left(x^{\prime },y^{\prime }\right)\cdot G\left(x+x^{\prime },y+y^{\prime }\right),\\ F\left(x,y\right)=\left\{\begin{array}{c} 1,\;-\epsilon \leq T\leq \epsilon \\ 0,\;\mathrm{other}, \end{array}\right. \end{array}\right.$$

where ϵ is the size of the soft margin of SVR and F is edge response map.

Then the response map T is split into two components which are represented as $\left\{z_{p1}\right\}$ and $\left\{z_{p2}\right\}$, according to the character of symmetry. Every component is also split into *n* parts and written as a set $z_{pi},i=1,2$. The combinations between the elements $\left\{z_{p1}\right\}$ and $\left\{z_{p2}\right\}$ are evaluated as follows:

(13) $$\begin{array}{cc} S_{side}\left(z_{p1}^{i},z_{p2}^{j}\right) & =Score_{side}\left(z_{p1}^{i},z_{p2}^{j}\right)\\ & =F_{sum}\left(z_{p1}^{i}\right)+F_{sum}\left(z_{p2}^{j}\right)\\ & -\sqrt{{\left(x_{i}-x_{j}\right)}^{2}+{\left(y_{i}-y_{j}\right)}^{2}}, \end{array}$$

where $z_{p1}^{i}$ is the *i*th part in the set $z_{p1}$; $z_{p2}^{j}$ is the *j*th part in the set $z_{p2}$; $F_{sum}\left(z_{p1}^{i}\right)$ is the sum of the $z_{p1}^{i}$ in the respone map.

The response strength of the side model $F_{sum}$ and the Euclidean distance between two elements are considered to be the evaluation metric. It is obvious that the grasping position algorithm is more likely to locate in two elements which provide a high response through the side model and shorter distance.

According to their evaluation scores in $S_{side}\left(z_{p1}^{i},z_{p2}^{j}\right)$, the largest m(m≤n) combination is obtained. All these combinations apply the operation of dot product with the root model R to obtain the combination with the maximum score as the grasping positions:

(14) $$S_{root}=Score_{root}\left(z_{k}\right)=max\left\{R\left(x,y\right)\cdot F\left(z_{k}\right)\right\}.$$

## 5. Grasping Strategy and Control

In this section, an autonomous grasping strategy and control laws of the grasping system are proposed to perform the aerial grasping task. The center of mass of the UAV with the manipulator changes when the robotic arm moves, it makes the UAV unstable. To achieve stable grasping performance of the aerial grasping system, the grasping process is divided into the approaching phase and the grasping phase. The main task of the approaching phase is to control the UAV quickly and stably reach above the target object. In the grasping phase, the UAV equipped with the 3-DOF robotic arm perform autonomous target grasping.

### 5.1. Approaching Phase

The approaching phase aims to guide the UAV to move the target object quickly. In this phase, the 3-DOF robotic arm remains stationary. The gimbal is controlled by the PD controller [29]. The controller of the UAV is designed according to the Lyapunovs second theory.

**Figure 4:**
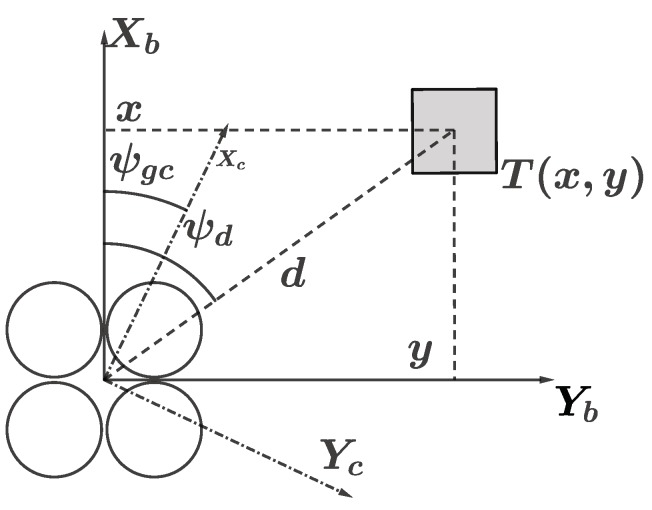
The position relationship between the aircraft and the target object on the two-dimensional plane.

The position relationship between the UAV and the target on the two-dimensional plane is shown in Figure 4, where four circles denote the UAV, whose position can be written as $P_{t}={\left[\hat{x},\hat{y}\right]}^{T}$. The position $P_{t}$ of UAV can be estimated by Equation (27). Let $\hat{d}$ be the estimation of the distance between the target object and the UAV, it can be calculated by

(15) $$\hat{d}=\sqrt{{\hat{x}}^{2}+{\hat{y}}^{2}}.$$

Let $\psi_{d}$ be the desired rotation angle of the yaw, it can be calculated by

(16) $$\psi_{d}=\arctan\frac{\hat{y}}{\hat{x}}.$$

Then the estimation of velocity $\dot{\hat{d}}$ and the angular velocity $\dot{\psi_{d}}$ can be written as:

(17) $$\left\{\begin{array}{c} \dot{\hat{d}}=\frac{1}{d}\left(\hat{x}\dot{\hat{x}}+\hat{y}\dot{\hat{y}}\right)=v_{x}\cos\psi_{d}+v_{y}\sin\psi_{d},\\ \dot{\psi_{d}}=\omega_{d}. \end{array}\right.$$

In real-world applications, there exists an error between the actual velocity and the desired velocity of the UAV. The error consists of two parts, one is the error between the desired linear velocity and the actual linear velocity in the horizontal direction $\epsilon_{v}$, while another is the angle error between the desired yaw angle and the actual yaw angle $\epsilon_{\psi }$. In addition, let $\epsilon_{d}$ denote the error between the actual distance and the desired distance. According to Figure 4, it can be obtained by:

(18) $$\left\{\begin{array}{c} \epsilon_{d}=\hat{d}-l,\\ \epsilon_{v}=v_{d}-v_{v}=\sqrt{v_{x}^{2}+v_{y}^{2}}-\sqrt{v_{rx}^{2}+v_{ry}^{2}},\\ \epsilon_{\psi }=\psi_{d}-\psi_{r}, \end{array}\right.$$

where $v_{rx}$ and $v_{ry}$ are the actual velocities of the UAV in the *X* and *Y* directions, respectively.

The time derivative of Equation (18) is

(19) $$\left\{\begin{array}{c} {\dot{\epsilon }}_{d}=\dot{\hat{d}}=v_{x}\cos\psi_{d}+v_{y}\sin\psi_{d},\\ {\dot{\epsilon }}_{v}={\dot{v}}_{d}-{\dot{v}}_{r}={\dot{v}}_{x}\cos\psi_{d}+{\dot{v}}_{y}\sin\psi_{d},\\ \dot{\epsilon_{\psi }}={\dot{\psi }}_{d}-{\dot{\psi }}_{r}=\omega_{d}, \end{array}\right.$$

where $\psi_{r}$ is yaw rotation angle and $\omega_{d}$ is the yaw angular velocity of the UAV.

In the approaching phase, the velocity $v_{x}$, $v_{y}$ and angular velocity $\omega_{d}$ of UAV are controlled to ensure that the distance error $\epsilon_{d}$, velocity error $\epsilon_{v}$ and angular error $\epsilon_{\psi }$ converge to zero. The control law of the UAV is designed as:

(20) $$\left\{\begin{array}{c} v_{x}=k_{1}\left(\epsilon_{d}+\epsilon_{v}\right)\cos\psi_{d}+\frac{v_{crx}\epsilon_{v}}{\epsilon_{d}+\epsilon_{v}},\\ v_{y}=k_{1}\left(\epsilon_{d}+\epsilon_{v}\right)\sin\psi_{d}+\frac{v_{cry}\epsilon_{v}}{\epsilon_{d}+\epsilon_{v}},\\ \omega_{d}=k_{2}\epsilon_{\psi }, \end{array}\right.$$

where $k_{1}$ and $k_{2}$ are coefficient less than zero, $v_{crx}$ and $v_{cry}$ are the actual velocities of the current moment of the UAV in the *X* and *Y* directions, respectively.

The stability of the system can be proved using Lyapunovs second theory. The Lyapunov function candidate can be formulated as:

(21) $$V\left(x\right)=\frac{1}{2}\left(\epsilon_{d}^{2}+\epsilon_{v}^{2}+\epsilon_{\psi }^{2}\right).$$

Please note that V(x)≥0 and V(x)=0 if and only if ${\left[\epsilon_{d}\;\epsilon_{v}\;\epsilon_{\psi }\right]}^{T}={\left[0\;0\;0\right]}^{T}$. The time derivative of V(x) is

(22) $$\begin{array}{cc} \dot{V}\left(x\right) & =\epsilon_{d}{\dot{\epsilon }}_{d}+\epsilon_{v}{\dot{\epsilon }}_{v}+\epsilon_{\psi }{\dot{\epsilon }}_{\psi }\\ & =\left(v_{x}\epsilon_{d}+{\dot{v}}_{x}\epsilon_{v}\right)\cos\psi_{d}\\ & +\left(v_{y}\epsilon_{d}+{\dot{v}}_{y}\epsilon_{v}\right)\sin\psi_{d}\\ & +\omega_{d}\epsilon_{\psi }. \end{array}$$

The acceleration of *X* and *Y* directions can be calculated by:

(23) $$\left\{\begin{array}{c} {\dot{v}}_{x}=v_{x}-v_{crx},\\ {\dot{v}}_{y}=v_{y}-v_{cry}. \end{array}\right.$$

Using Equations (20), (22) and (23), we simplify the time derivative of V(x) as

(24) $$\left\{\begin{array}{c} \dot{V}\left(x\right)=k_{1}{\left(\epsilon_{d}+\epsilon_{v}\right)}^{2}+k_{2}\epsilon_{\psi }^{2},\\ k_{1},k_{2}\leq 0. \end{array}\right.$$

Equation (24) ensures that $\dot{V}\left(x\right)\leq 0$, while $k_{1},k_{2}\geq 0$. Thus, the control system is Lyapunov stable with the designed control law.

### 5.2. Grasping Phase

When the pitch angle of the gimbal is $90^{\circ }$, it means that the UAV is just above the target. The grasping phase works. At this phase, we control the height of the UAV and the robotic arm to grasp the target object vertically.

**Figure 5:**
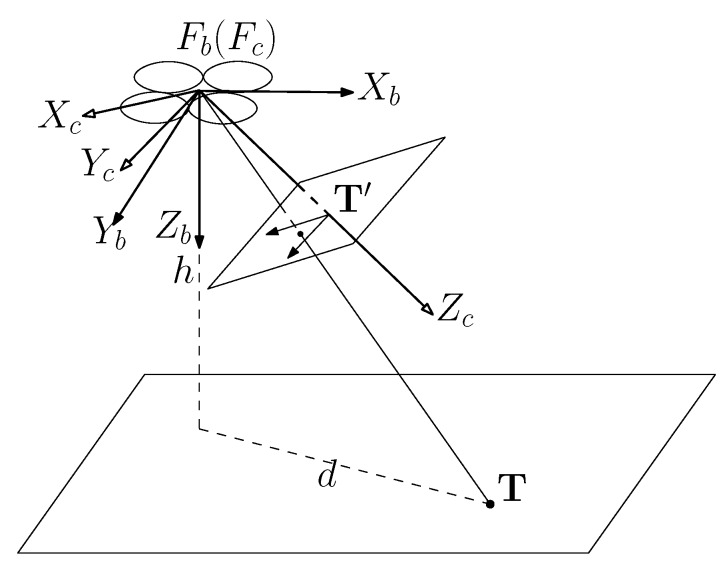
The relationship among the UAV, the camera and the target.

Figure 5 shows the relationship among the UAV, the camera and the target, where $F_{b}$ denotes the body frame of UAV with axes $X_{b}$, $Y_{b}$ and $Z_{b}$, and $F_{c}$ denotes the camera’s reference frame with axis $X_{c}$, $Y_{c}$ and $Z_{c}$. The rotation matrix $R_{bc}$ from $F_{c}$ to $F_{b}$ can be calculated by:

(25) $$R_{bc}=R_{wb}R_{wc}^{T},$$

where $R_{wb}$ is a transformation matrix from the world frame to the body frame; $R_{wc}$ is a transformation matrix from the world frame to the camera’s reference frame.

The position of target object in $F_{b}$ can be calculated by:

(26) $$T=R_{bc}^{T}{\mathrm{PK}}^{-1}A^{T},$$

where $T=\left[x_{b},y_{b},z_{b}\right]$ is the position of target object in $F_{b}$; K is the intrinsic matrix of the camera; P is the permutation matrix; $A=\left[u,v,1\right]$ indicates the position of the target on the image plane.

According to standard pinhole imaging model, the position of target object $P=\left[x,y,z\right]$ can be estimated by:

(27) $$\left\{\begin{array}{c} \hat{x}=\frac{h}{z_{b}}x_{b},\\ \hat{y}=\frac{h}{z_{b}}y_{b},\\ \hat{z}=0, \end{array}\right.$$

where *h* is the height of the UAV. It can be detected by the ultrasonic sensor.

PID controller is used to control the position and height of the UAV. The position error can be calculated by:

(28) $$\left\{\begin{array}{c} e_{x}=x_{b}-0,\\ e_{y}=y_{b}-0, \end{array}\right.$$

where $e_{x}$ and $e_{y}$ are error in *X* and *Y* directions respectively, $x_{b}$ and $y_{b}$ are position of target in $F_{b}$ respectively. The desired height of the UAV can be calculated by:

(29) $$h_{d}=h-l,$$

where $h_{d}$ is the desired height of the UAV, *l* is the maximum distance of the robotic arm, and *h* is the height of the UAV. It can be detected by the ultrasonic sensor.

The joints of the arm are controlled to keep the robotic arm vertical. The gripper at the end of the robotic arm grasp the target object when the UAV hovers at the desired height.

## 6. Experimental Results

To verify the autonomous vision-based aerial grasping system, extensive flight experiments are performed in outdoor environments. First, the performance of the target object detection and recognition scheme is verified and analyzed. Second, the elapse time and performance of the grasping position detection algorithm is examined. The designed control laws are then verified by the simulation and real-world flight experiments. Finally, experimental results of the autonomous vision-based aerial grasping in real-world are presented.

### 6.1. Experimental Setup

**Figure 6:**
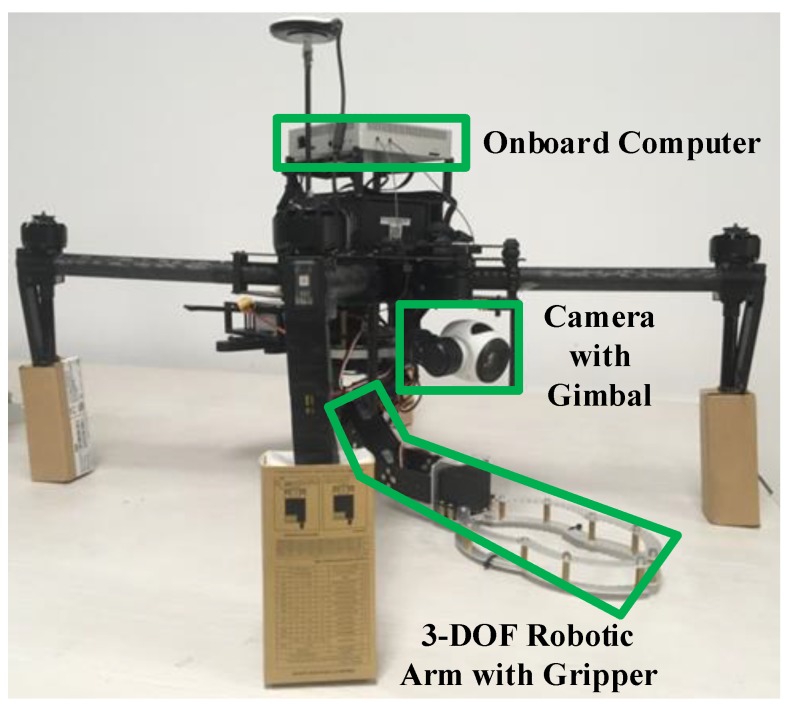
The UAV experiment platform with 3-DOF robotic arm.

A DJI Matrice 100 UAV is used as an experiment platform, as shown in Figure 6. Airborne equipment includes a DJI Manifold embedded Linux computer (NVIDIA Tegra TK1 processor, an NVIDIA 4-Plus-1 quad-core A15 CPU of 1.5 GHz), a GPS receiver, a 3-DOF robotic arm, a monocular Zenmuse X3 gimbal camera, a barometer, an Inertial Measurement Unit (IMU) and a DJI Guidance visual sensing system.

### 6.2. Object Detection and Recognition Experiment

The purpose of this experiment is to test the performance of the computationally efficient object classification correlation filter based on ridge regression. The dataset used in this experiment is the extended ETHZ dataset [34] that is extended from five classes to six classes. The new dataset includes six classes, of which the classes toy cars is entirely and newly collected by ourselves. The sample number of each category is shown in Table 1.

**Figure 7:**
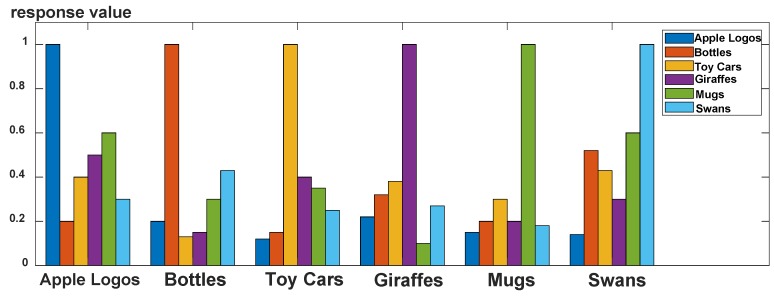
Performance Comparison of Correlation Filter Target Classifier Based on Ridge Regression.

The reason for adopting the small dataset is that the KCF algorithm learning module has the feature of increasing samples through circular displacement. The evaluation criteria of the experiment is the average correlation value of the model to the positive and negative samples after performing 10 times a 5-fold cross validation for each category model. Figure 7 shows the experiment results.

As shown in Figure 7, each class model obtained by training has a higher response value to the positive samples in the test set, and the response value is basically much larger than the response value to other categories. It shows that this type of classifier has better classification performance for simple objects. At the same time, correlation detection is performed in the frequency domain. Thus, its detection operation time is also fast with the help of fast Fourier transform (FFT). In the experiment, the average detection time of each sample is 0.02s.

### 6.3. Grasping Position Detection Experiment

The purpose of this experiment is to verify the accuracy and elapse time of the grasping position detection algorithm. The dataset is from the research of [35]. The resolution of the root model is set to 80×80×31. Furthermore, separating the resized image into two components for training the side model. Therefore, the resolution of the side model is set to 80×40×31. The results of the grasping position detection experiment is shown in Table 2 and Table 3. As shown in Table 2, the accuracy of the grasping position model, which is the combination of side model and root model, is acceptable.

As shown in Table 3, the algorithm of grasping position detection is real-time within the range of 0.3 million. The largest computational cost is to use the side model to detect the shape of object. Therefore, it is necessary to restrict the resolutions of input image for real-time grasping position detection.

### 6.4. UAV Control Experiments

#### 6.4.1. Simulation Experiments

**Figure 8:**
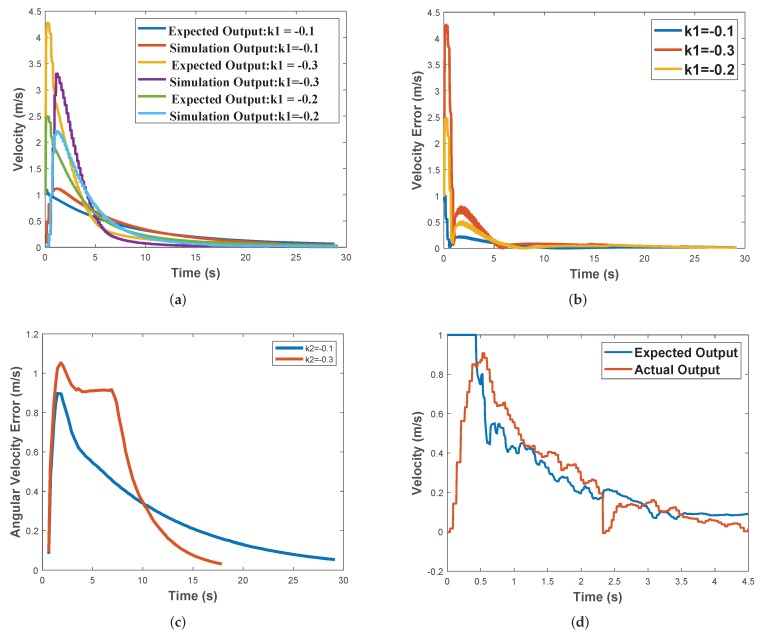

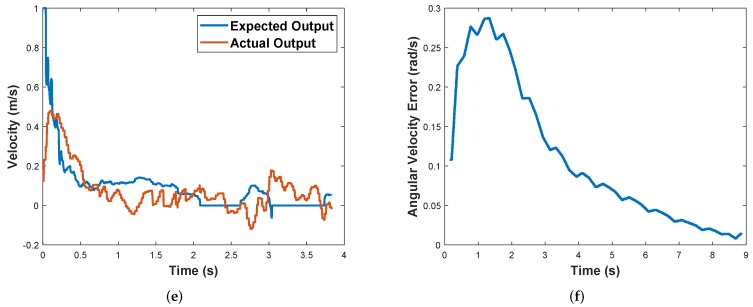
(**a**) Simulation results in the x direction; (**b**) The error between the desired velocity and the simulation velocity in the X direction simulation; (**c**) The error between the desired yaw angle and the simulation angular velocity; (**d**) Experimental results in the x direction in the real-world flight tests; (**e**) Experimental results in the y direction in the real-world flight tests; (**f**) The error between the desired angular velocity and the yaw angular velocity in the real-world flight tests.

The DJI Assistant 2 aircraft simulation platform is used in this simulation experiment. The experiment design is as follows: control law (20) is verified where the adjustable parameters are set as $k_{1}$ and $k_{2}$. In this experiments, three groups of value are set for simulation and real-world flight experiment. According to the symmetry property of quadrotor aircraft, the test of the parameters just needs to test one direction. The test direction of this experiment is X direction. In the experiment setting, we set $k_{1}=-0.1$, $k_{1}=-0.2$, $k_{1}=-0.3$ and the flight distance is 10 m. The simulation results are shown in Figure 8a–c.

According to Figure 8a,b, we can see that the adjustment trend of the control law become more obvious when we set higher parameter value. The velocity of the UAV gradually converges to the desired value, and the errors between the desired values and the simulated values also gradually converges.

The parameter $k_{2}$ is adjusted in simulation by the same method. We set $k_{2}=-0.1$, $k_{2}=-0.3$ and the desired of UAV yaw angle is $90^{\circ }$. The error of the simulation angular velocity is shown in Figure 8c.

Similar to the error of the velocity control, when the parameter value is larger, the initial desired angular velocity of the UAV controller is larger as well. As the rotation angle reaches the target angle, it gradually converges. The greater the parameter is, the faster the convergence velocity is.

#### 6.4.2. Experiments of Flight Tests

In the flight experiments, we select two parameters $k_{1}=-0.2$ and $k_{2}=-0.3$. The maximum speed of the aircraft is restricted to 1m/s, and the attitude data of the UAV are measured by the onboard IMU module. The flight experimental results are shown in Figure 8d–f.

The experimental results show that the actual velocity values converge to the desired velocity in 0.5s and follows the desired velocity very well. The error curve of the yaw angular velocity in actual flight test is shown in Figure 8f. The yaw angle errors decrease gradually from a relatively large value to the desired zero value.

### 6.5. Autonomous Aerial Grasping Experiments

**Figure 9:**
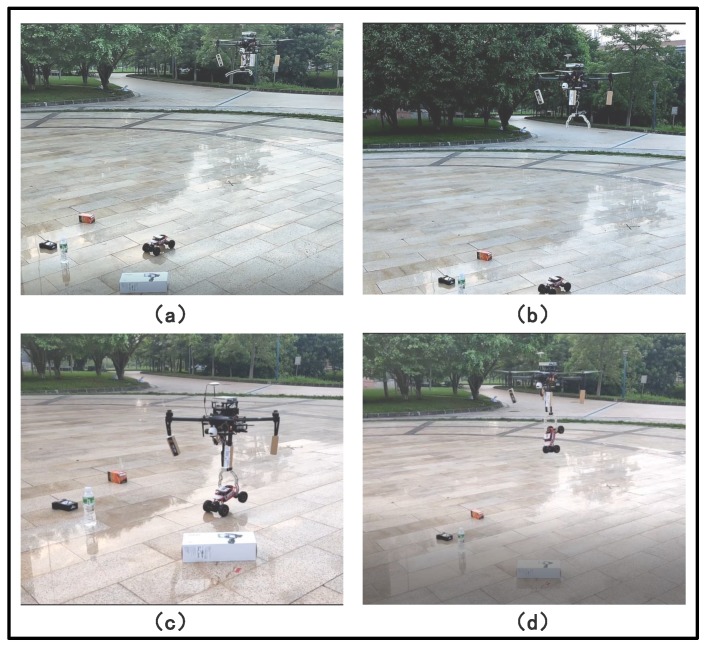
UAV autonomous grasping system test. (**a**) The approaching phase; (**b**,**c**) the grasping phase; (**d**) the UAV to complete the grasping task and ascent to the specified height.

The proposed algorithms and the developed aerial grasping system are systematically investigated in flight experiments. In the experiments, as shown in Figure 9, the target object, a toy car, will be detected among some other objects within the visual view of the gimbal camera. The parameters of PID controller is shown in Table 4. Snapshots of the grasping process are illustrated in Figure 9, where Figure 9a is the approaching phase, Figure 9b,c are the grasping phase, and Figure 9d is the UAV to complete the grasping task and ascent to the specified height. A demo video of the proposed aerial grasping system in outdoor environments can be seen in the supplementary video.

Limitation and discussion: to examine the grasping performance, 10 successive grasping experiments are conducted in outdoor environments. The achieved success rate of the aerial grasping of the toy car is 50%. Vision-based autonomous aerial grasping is a systematically work, and the performance of each part of the visual perception as well as control of the UAV and the robotic arm will affect the grasping performance. For the visual perception part, according to Figure 7, the trained classifier has good performance; however, the accuracy of the grasping point estimate algorithm is 74.1%. It is noted that in the grasping phase, there is a lag in the position control of the UAV. Moreover, mechanical instability and low response of the robotic arm and the end gripper also deteriorate the grasping performance. In future work, the grasping points estimate will be further studied, and the mechanical design of the robotic arm will also be considered to improve the grasping performance.

## 7. Conclusions

In this paper, an autonomous vision-based aerial grasping system for a rotorcraft UAV is presented, where the target object is fully autonomously detected and grasped. The proposed visual perception and control strategies are systematically studied. An efficient object detection and tracking method is addressed to improve the KCF algorithm. A grasping positions estimate of the target object is proposed based on the edge and root model thereof, to increase the grasping success rate. Based on the estimated relative position between the target object and the UAV as well as the grasping points of the target object, control laws of the UAV and the robotic arm are proposed to guide the UAV to approach to and grasp the target. The visual perception and control are implemented on an onboard low-cost computer. Experiment results illustrate that the proposed autonomous vision-based aerial grasping system achieves stable grasping performance. In future work, the grasping points estimate will be further studied to improve the estimate accuracy. Mechanical design of a stable and light weight robotic arm will be considered. Autonomous grasping of a moving target object is also worth investigation.

## Figures and Tables

**Table 1:** The category and sample size of extended ETHZ dataset.

Category	Apple Logos	Bottles	Toy Cars	Giraffes	Mugs	Swans
Quantity	44	55	42	91	66	33

**Table 2:** The results of the grasping position detection algorithm.

Set	Number of Samples	Within Soft Margin
Training Set	1125	1006 (89.4%)
Positive (Test)	864	640 (74.1%)
Negative (Test)	719	267 (37.2%)

**Table 3:** The elapse time of the grasping position detection algorithm.

Resolution	S	R	T
140×60	0.001 s	0.001 s	0.002 s
265×120	0.021 s	0.005 s	0.026 s
493×240	0.134 s	0.006 s	0.14 s

Note: S means the elapse time of the side model. R means the elapse time of the root model. T means the total of the elapse time.

**Table 4:** The parameters of the PID controller.

Parameters	P	I	D
Yaw	0.13	0	0.05
Pitch	0.08	0	0.03
UAV	2.3	0.1	1.28

Note: Yaw and Pitch imply the yaw and pitch angle control of the gimbal, respectively. UAV implies the position control of the UAV.
